# Supplementary figures and images for: Antineoplastic kinase inhibitors: A new class of potent anti-amoebic compounds
Source: PLoS Negl Trop Dis. 2021 Feb 8;15(2):e0008425. doi: 10.1371/journal.pntd.0008425 (PMC7895358; doi:10.1371/journal.pntd.0008425)

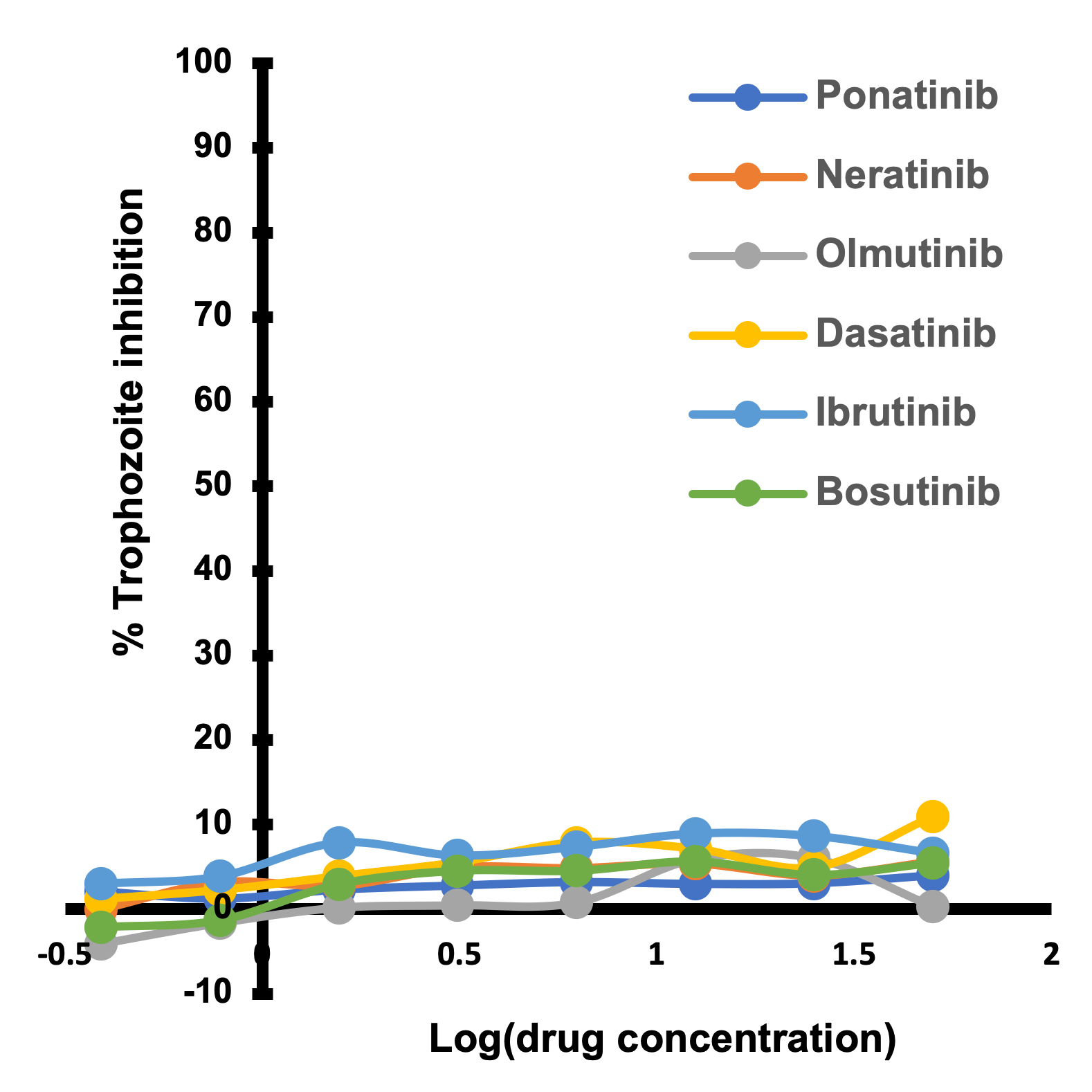

Supplement: S1 Fig — All drugs were tested at a serially-diluted range of concentrations. Cell viability measured at T = 0. (TIF) [file pntd.0008425.s001.tif]
