# Supplementary material for: Antineoplastic kinase inhibitors: A new class of potent anti-amoebic compounds
Source: PLoS Negl Trop Dis. 2021 Feb 8;15(2):e0008425. doi: 10.1371/journal.pntd.0008425 (PMC7895358; doi:10.1371/journal.pntd.0008425)
Supplement: Attachment — Submitted filename: Response_to_reviewers.docx [file pntd.0008425.s008.docx]

Dear Editor and Reviewers,

We, the authors of *"Antineoplastic kinase inhibitors: a new class of potent anti-amoebic compounds"* would like to thank the reviewers for their valuable feedback regarding this manuscript and will here try to address each point and concern. In addition, several edits to the manuscript text have been made and submitted along with this letter.

**Reviewer #1: The EC50 of the drugs was coming out in submicromolar ranges. The authors should use the concentration of the drug concentration near the EC50, why much higher concentration (10uM) was used for survival assays?**

Author response: This is an interesting question posed by Reviewer #1. We have edited the manuscript text to clarify this point and hope the following will also clarify our reasoning: Typically for most anti-parasitic drugs the therapeutic effect occurs at concentrations well above the EC50 value. Other than the assays specifically designed to determine the EC50 values of the drugs, most of the other survival assays in this study were designed using excess concentrations of drugs specifically in order to ensure complete killing of the parasite to a therapeutically relevant level. In these other assays information related to the concentration-dependent effects of the drugs was not desired, but rather other information such as timing or parasite killing vs. growth inhibition etc.

We have edited the text to reflect this as follows: (Line 358) *“A concentration of 10µM was used for all drugs in order to ensure maximal and unambiguous inhibitory effects against the trophozoites.”*

**Reviewer #1: Why different concentration of drugs was used for different assays- Page 21 (line 352) and page 23 (line 406)**

Author response: This is a good question, and hopefully the following will clarify this point: Both of these concentrations were chosen to be in large excess of the amount needed for complete parasite killing. The first experiment mentioned by Reviewer #1 (line 352) included the drugs dasatinib, ibrutinib, bosutinib as well as ponatinib, neratinib, and olmutinib, whereas the second (line 406) only included ponatinib, neratinib, and olmutinib. The higher concentration used in the first experiment corresponds to the relatively higher EC50 values of dasatinib/bosutinib/ibrutinib, whereas the lower concentration used in the second experiment corresponds to the higher potency and lower EC50 values of ponatinib/neratinib/olmutinib.

**Reviewer #1: Page16 (line 260-261) please mention reference and if there is not then what is the basis taken for the cutoff value chosen as 15?**

Author response: The cutoff value of 15 was chosen somewhat qualitatively. A naturally occurring gap in activity scores exists between drugs scoring 15 and the next lowest-scoring drugs (~11.5) and the few drugs scoring below that level possess only very few and very weak activities against the target profile proteins.

We would like to thank Reviewer #1 for alerting us to possible confusion stemming from this, and have edited the text to address the issue as follows: (Line 262) *“This cutoff value was chosen based on a naturally occurring gap between drugs with a score of 15 and the next-lowest scoring drugs, with the latter possessing only few and very weak activity scores against any of the target profile proteins.”*

**Reviewer #1: Fig. 8F shows that the effect of the drug is maximum at 36 hours, then why calculations were done with reference to effect of the drug at 48 hours?**

Author response: We would like to thank the reviewer for this observation and hope the following will satisfactorily clarify this point: Calculations for this figure were made relative to the 48hr exposure time readings in order to allow for comparison across drug treatments after different exposure times of the parasite to each drug. (as discussed in lines 420-423) Effectively, calculating in this way allowed us to detect the inhibition maximums observed at 36hr for some of the drugs. Only 2 out of the 4 drugs tested displayed maximum inhibition after 36hr, hence why we chose to calculate relative to the final timepoint.

**Reviewer #1: Please mention time for calculating EC50 values in figure legends for fig. 1 and 6**

Author response: We would like to thank Reviewer #1 for this suggestion, and the manuscript has now been modified accordingly.

**Reviewer #1: Figure 6 is showing that there is almost 100% trophozoite inhibition when drugs are added but in figure 7 100% inhibition is not shown. please mention the reason for that.**

Author response: This is a great observation from Reviewer #1, and definitely a point worth clarifying. The 100% inhibition value in Figure 6 was calculated relative to the positive control drug, metronidazole. Metronidazole was chosen as the 100% inhibition control based on its status as the current gold standard of therapy for the *E. histolytica* parasite, as described in the manuscript. The values in figure 7 are not calculated as percentage inhibition relative to the positive control as in Figure 6. They are instead calculated relative to the assay signal obtained from a fixed, known number of cells. In fact, as reviewer #1 has noticed, these experiments taken together indicate that either metronidazole may not kill 100% of trophozoites within 48hr.

We have edited the text to include the following: (Line 366) *“Interestingly, none of the drugs tested, including metronidazole, completely reduce the estimated cell number to zero. This could possibly be due to either a small number of genuine surviving cells, or to dying cells which still contain measurable ATP.”*

**Reviewer #1: Resolution for Figure 5 and 8 is very bad, nothing is clear.**

Author response: We agree with Reviewer #1 and thank them for bringing this issue to our attention. Upon further investigation it appears that in order to access the figure images in their true resolution they must be downloaded using the link at the top of each figure page in the .pdf file of the manuscript provided by the journal. We will also contact the journal to ensure that the full-resolution images will be used in the final print.

**Reviewer #1: I was not able to access supplementary fig 1.**

Author response: We apologize to the reviewer for this issue and have investigated the problem further. We were able to access supplementary figure 1 by clicking the “Click here to access/download supplementary information” button on the .pdf file provided by the journal. Possibly Reviewer #1 may need to re-download this file and try again?

**Reviewer #1: These are all AKI drugs, what can be the effect of these drugs when given to non cancer patients..**

Author response: We would like to thank the reviewer for this insightful observation and have expanded upon this point in the discussion section of the manuscript. (Line 489) While studies of these types of AKI drugs in healthy volunteers instead of cancer patients are somewhat rare, we were able to find publications detailing the effects of Olmutinib as well as a few related investigational BTK inhibitors. All of these were described as generally “safe and well-tolerated,” similarly to when they have been tested in cancer patients. Also similarly to studies in cancer patients, the most common adverse effect was diarrhea, an aspect we briefly discussed in the manuscript.

We have edited the text to reflect this as follows: (Line 489) *“This has been observed both in studies of cancer patients and of healthy volunteers, in which these drugs are nevertheless generally described as “safe and well-tolerated.””*

**Reviewer #1: As these drugs target kinases that have homologs present in bacteria, amoeba and animals what can be the effect of these drugs on intestinal microbes?**

Author response: This is a very interesting question, and one likely related to Reviewer #1’s previous point. The literature on this topic indicates that AKI drugs have the ability to disrupt the composition of the gut microbiome, and that this disruption is associated with patients experiencing diarrhea as an adverse effect. We have added a statement about this in the manuscript text along with the relevant citations.

(Line 493) *“This could possibly be due to disruption of the intestinal microbiome by the AKI drugs, which has been previously reported.”*

**Reviewer #1: Interestingly, most of these inhibitors are tyrosine kinase inhibitors. And amoeba genome has 372 kinases and about 250 phosphatases. There are several studies, any disturbance in the kinase-phosphatase signalling pathway could be fatal for the organism (Ahmad et al., 2020, Mansuri et al., 2014, 2016). Then why only few inhibitors are effective and others are not? Can the authors predict or perform, which kinases are inhibited? Which family of kinases are more crucial?**

Author response: We would like to thank the reviewer for this excellent question. We were very interested in this as well, and tried to partially address it in Figure 5, which unfortunately was rendered illegible by the low-resolution issue. We have taken steps to correct this issue with Figure 5 and hope it will clarify this point. In our analysis we searched the proteome of *E. histolytica* for potential orthologs of the human proteins known to be targeted by the potent AKI drugs such as ponatinib, etc. We found a cluster of putative *E. histolytica* kinases which have good homology to several of these human kinases. We conclude that it is likely that this group of *E. histolytica* kinases are the cellular targets of the drugs we discuss in this manuscript, and investigation of this possibility forms a key aspect of our goals for future studies.

Thank you,

Conall Sauvey

University of California - San Diego

Skaggs School of Pharmacy and Pharmaceutical Sciences
